# A comparison of in-house and shared RapidPlan models for prostate radiation therapy planning

**DOI:** 10.1007/s13246-022-01151-1

**Published:** 2022-09-05

**Authors:** E. Claridge Mackonis, J. Sykes, N. Hardcastle, A. Espinoza, A. Brown, G. Perez, B. Evans, H. Sheehan, A. Haworth

**Affiliations:** 1 Department of Radiation Oncology, Chris O’Brien Lifehouse, Camperdown, NSW Australia; 2 Blacktown Cancer and Haematology Centre, Sydney West Cancer Network, Sydney, Australia; 3 Department of Physical Sciences, Peter MacCallum Cancer Centre, Melbourne, Australia; 4 Westmead Hospital, Crown Princess Mary Cancer Centre, Westmead, Australia; 5 Radiation Oncology, Central West Cancer Care Centre, Orange, Australia; 6 Institute of Medical Physics, University of Sydney, Sydney, Australia

**Keywords:** RapidPlan, Knowledge-based planning (KBP), Prostate, Radiation oncology, Shared models

## Abstract

Knowledge-based planning (KBP) can increase plan quality, consistency and efficiency. In this study, we assess the success of a using a publicly available KBP model compared with developing an in-house model for prostate cancer radiotherapy using a single, commercially available treatment planning system based on the ability of the model to achieve the centre’s planning goals. Two radiation oncology centres each created a prostate cancer KBP model using the Eclipse RapidPlan software. These two models and a third publicly-available, shared model were tested at three centres in a retrospective planning study. The publicly-available model achieved lower rectum doses than the other two models. However, the planning-target-volume (PTV) doses did not meet the local planning goals and the model could not be adjusted to correct this. As a result, the plans most likely to satisfy local planning goals and requirements were created using an in-house model. For centres without an existing in-house model, a model created by another centre with similar planning goals was found to be preferred. Variations in local planning practices including contouring, treatment technique and planning goals can influence the relative performance of KBP. The value of publicly available KBP models could be enhanced through standardisation of planning goals and contouring guidelines, providing information related to the planning goals used to create the model and increased flexibility to allow local adaptation of the KBP model.

## Introduction

Knowledge-based planning is a technique where knowledge from historical planning data is combined with patient anatomical information to inform achievable plan quality for the individual patient. The introduction of knowledge-based planning (KBP) for an automated, individualised approach for optimising modulated radiotherapy has been shown to increase plan quality and decrease inter-planner variation [1, 2]. Its implementation into the Eclipse treatment planning system, under the name RapidPlan (Varian Oncology Systems, Palo Alto, Ca, USA) has been validated in numerous studies [3–7]. RapidPlan uses dose-volume histogram (DVH) predictions based on previous plans to create individualised optimisation criteria.

The New South Wales RapidPlan Consortium was formed in late 2018 to investigate possible benefits which could be gained through collaboration between centres using RapidPlan. Whilst multiple publications have reported methods for optimising RapidPlan models for local use, few publications have reported potential benefits of model sharing across multiple institutes.

Schubert et al. [8], representing the German RapidPlan Consortium, distributed a model from a single centre to seven separate institutions and found that the model produced clinically acceptable plans at all centres. However, it was postulated that further fine tuning of the model at the local site may be beneficial given the differences between institutions in terms of contouring, planning techniques and planning goals. Ueda et al. [9] investigated the differences between models produced independently at 5 institutions and concluded that sharing models would require that the plan designs used for the DVH estimate match those used in the institution receiving the model. In contrast, rather than sharing models, the Victorian Public Sector RapidPlan Group created a prostate model jointly between eight separate radiotherapy centres [10]. When comparing the retrospective, manually produced plans with the Group-model generated plans, they found a general improvement in organ-at-risk doses and dose homogeneity. However, they also found that the introduction of a common automated model did not reduce the range of OAR doses (ie the minimum to maximum values) within each centre.

With the growing evidence of the benefits of using RapidPlan, many centres are looking to implement KBP. However, limited guidance on the best and most efficient ways to do this is provided in the literature. To investigate this, we designed a study to ask the following question: Is it better to develop an in-house model, use an externally developed model or modify an externally developed model? To answer this question, three centres evaluated two locally generated models and one publically available model. Each centre used the models to generate plans for a set of previously treated patients. Each centre evaluated the generated plans according to their standard (in-house) qualitative and quantitative planning goals.

## Methods and materials

RapidPlan, implemented in the Eclipse Treatment Planning System (Varian Oncology Systems, Palo Alto, USA), was used for KBP model generation. For this study, three radiation therapy centres were enrolled, referred to as Centre A, B and C. Centre C had no prior KBP experience or local models. They aimed to determine from this study if an existing model would satisfy their requirements or whether they should instead create their own model. Centres A and B had existing prostate models which were included in the testing, referred to as Model A and Model B, respectively. Centres A and B aimed to determine whether an external model would better satisfy their planning requirements compared with their in-house models.

In addition to the in-house models from Centres A and B, the University of California, San Diego (UCSD) ‘UCSD Prostate’ [11] model was included in the testing as an example of a freely-available, shared model. Information regarding the 3 models is given in Table 1. Model A and B both include multiple target structures to allow the higher doses to be constrained to the central portion of the planning target volume (PTV).

**Table 1:** Details of the initial KBP models

Model	Model A	Model B	UCSD
From	Centre A	Centre B	Downloaded from UCSD
Version	13.7.16	13.6.23	13.6.23
Number of patients	92	41	105
Number of targets	2	3	1
OARs	Bladder	Bladder	Bladder
	Femoral heads	Femoral heads	Left femur
	Rectum	Rectum	Right femur
		Small bowel	Rectum
			Penile bulb
Intended use	Intact prostate or prostate bed, VMAT technique	Intact prostate ± seminal vesicles, VMAT technique	Prostate, VMAT^a^

^a^The intent of this model is not explicitly stated, however the model is named ‘Prostate’ and ‘2-arc VMAT’ is listed as the beam configuration

At each of the three centres, 19–20 consecutive patients were selected after excluding those who would not normally be planned with a standard beam configuration, for example patients with bi-lateral hip prostheses. Each centre planned their selected cohort using each of the three KBP models. Planners were asked to use the prescription dose, beam configuration and energy they would normally use in their centre. All centres chose to plan using volumetric modulated arc therapy (VMAT). Patients were planned with a single-click optimisation with no adjustments made by the planner. Plans were completed in Eclipse V13 using the AAA algorithm (Centres A and B) or Eclipse V15 using the AXB dose-to-medium algorithm (Centre C). In line with current practise at each centre, Centre A plans used 10MV while plans at Centres B and C used 6MV.

In addition to the three initial models, Centres A and B created and applied an edited version of the UCSD model, labelled as UCSD Edit. This new model was created by adjusting the objectives, including both dose level and priority, in the UCSD model to better satisfy the planning goals of those centres.

Dose volume histogram (DVH) data were then extracted using the Eclipse Scripting API and compared to the centres’ own prostate planning goals for each patient’s dose prescription. The planning goals of the three centres for a 78 Gy prostate plan are presented in Table 2. In addition, the planning goals from the eviQ ‘Prostate adenocarcinoma definitive EBRT conventional high risk’ protocol [12] are listed for comparison. eviQ does not provide recommendations on target coverage. In addition to the values listed in Table 2, eviQ quotes planning goals from trial protocols. These have not been included as they often differ from the eviQ recommendations. The eviQ protocol also includes constraints for the penile bulb, small intestines and large intestines. These are not listed as none of the centres in this study contour these organ-at-risk (OAR) structures.

**Table 2:** Prostate planning goals for 78 Gy prostate plans

Volume	DVH value	eviQ (12)	Centre A	Centre B	Centre C
CTV	V100%		> 99%		(> 99%)
PTV	V100%			> 99%	
	V95%		> 99%	> 99%	> 98%
	D1cc		< 107%		
	D2%				< 107%
	D99%				> 100%
	V107%			< 5%	
	Mean			> 102.4%	
	Mean			< 104.6 (104.4)%	
Bladder	V40	50%	50%		50%
	V50	50%	50%		
	V55			50%	
	V65	25%			25%
	V70		20%	30%	
	V78		5 cm^3^		
	V80		0.1 cm^3^		
Rectum	V40	35%	50%	60% (50%)	35%
	V60		35%	40% (35%)	
	V65	17%		25%	17%
	V70		20%	25% (20%)	
	V75	10%	10%	(15%)	5%
	V78		2cm^3^		
Femoral heads	V30		60%		
	V35	100%		100%	
	V45	60%			
	V50		5%	5%	5%
	V60	30%			

Values in brackets indicate goals which are desirable but not required

To allow comparison between the models as well as between the centres, the following dosimetry parameters were evaluated: PTV V95% and D1cc, bladder V40Gy, V50Gy and V65Gy, rectum V40Gy, V65Gy and V75Gy and femoral heads V50Gy, where VxxGy refers to the volume receiving xx in Gy. For these parameters, the mean values for each centre were calculated and these data presented in graphical format for comparison.

**Fig. 1:**
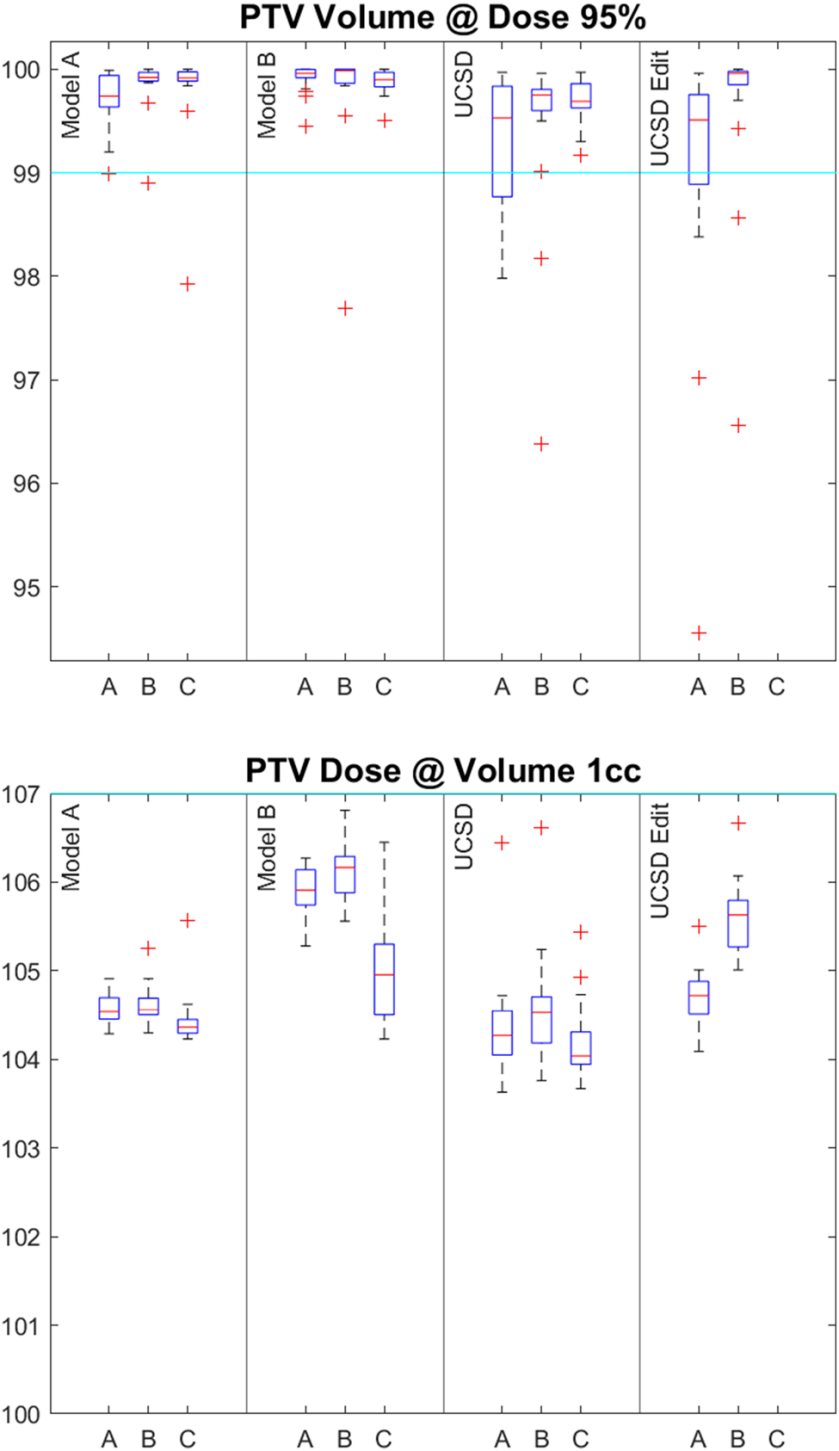
**a** The volume of PTV receiving 95% of the prescription dose (V95%) for the three centres (A, B, C) and for the four models (Model A, Model B, UCSD and UCSD edit), shown as a percentage. The cyan line indicates the eviQ acceptable level. **b** Percentage dose received by 1 cc of PTV (D1cc) for the three centres (A, B and C) and for the four models (Centre A, Centre B, UCSD and UCSD edit)

**Fig. 2:**
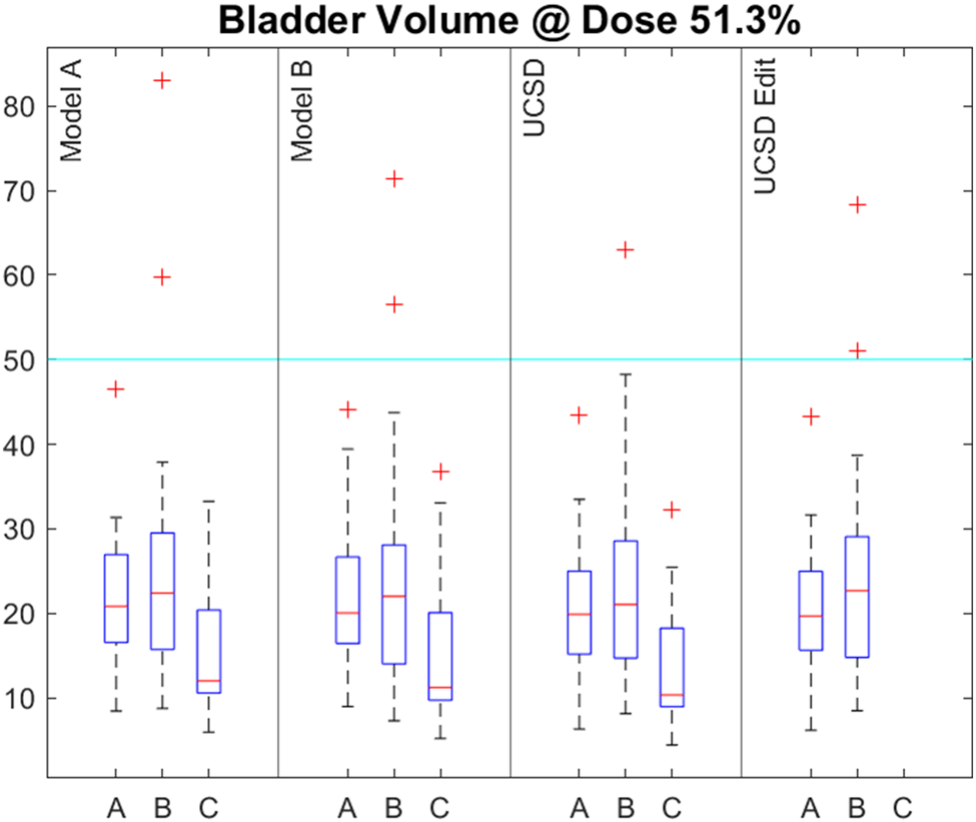
The percentage of bladder receiving a dose of 51.3% or more (equivalent to V40Gy for a 78 Gy prescription) for the three centres (A, B, C) and for the four models (Centre A, Centre B, UCSD and UCSD edit). The cyan line indicates the 78 Gy prostate eviQ acceptable tolerance

**Fig. 3:**
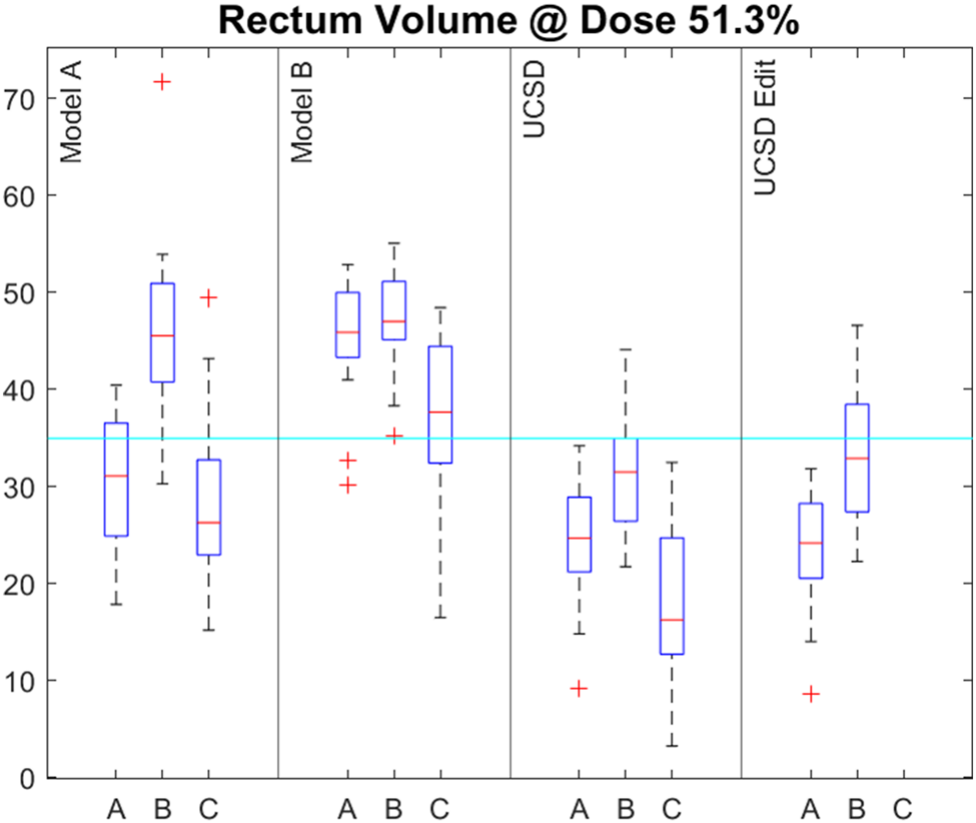
The percentage of rectum receiving a dose of 51.3% or more (equivalent to V40Gy for a 78 Gy prescription) for the three centres (A, B, C) and for the four models (Centre A, Centre B, UCSD and UCSD edit). The cyan line indicates the 78 Gy prostate eviQ acceptable tolerance

Statistical comparisons of the results were performed using Matlab™ with an unpaired 2-sample t-test used where *P* < 0.05 indicates significance in the difference of the mean values. The boxplots produced (Figs. 1, 2 and 3) to compare results display the interquartile range as a blue box with the median indicated by a red line and outliers shown as red ‘ + ’ symbols.

Each centre was asked to provide feedback regarding the performance of the RapidPlan models and which model(s) that centre intended to use in the future.

## Results

### Overall model performance

Considering the plans against all the local planning goals at each centre, as shown in Table 3, all models gave similar results. Centres A and C found Model A more likely to meet the planning goals while the edited UCSD model resulted in the highest proportion of planning goals met for Centre B.

**Table 3:** The average percentage of achieved local planning goals

	Model A	Model B	UCSD	UCSD edit
All local planning goals				
Centre A	80 ± 10%	72 ± 13%	76 ± 9%	76 ± 9%
Centre B	87 ± 3%	87 ± 3%	88 ± 2%	94 ± 6%
Centre C	92 ± 10%	88 ± 10%	86 ± 5%	
PTV local planning goals				
Centre A	75 ± 8%	99 ± 6%	57 ± 14%	57 ± 14%
Centre B	66 ± 4%	66 ± 4%	65 ± 5%	85 ± 16%
Centre C	93 ± 14%	97 ± 10%	67 ± 0%	
OAR local planning goals				
Centre A	81 ± 13%	64 ± 17%	82 ± 10%	82 ± 10%
Centre B	98 ± 5%	98 ± 5%	100 ± 0%	100 ± 2%
Centre C	91 ± 13%	84 ± 14%	94 ± 7%	

The values in the table are the average of the plans ± 1 standard deviation of these values

### PTV model performance

Model B meet a higher proportion of PTV planning goals at Centres A and C, while the UCSD edit Model met the higher proportion of PTV metrics at Centre B. Figure 1 compares the PTV V95% and D1cc data, showing the 3 centres and the 3 or 4 different models used at each centre. Note that the UCSD Edit models in the presented graphs are unique to each centre based on the changes made by that centre. At Centres B and C, all models generally achieved V95% greater than 99%, whereas at Centre A, only the Models A and B consistently achieved this objective. Comparing PTV V95% between centres, the results from Centres B and C do not show a significant difference between any of the models. The Centre A data are significantly different from Centre C for the UCSD model (*P* = 0.007) and also from Centre B for Model A (*P* = 0.048). Figure 1b shows the PTV D1cc for all centres. The 107% objective was meet by all plans. However, all centres found the mean D1cc to be higher with Model B (*P* < 0.001).

### OAR model performance

**Fig. 4:**
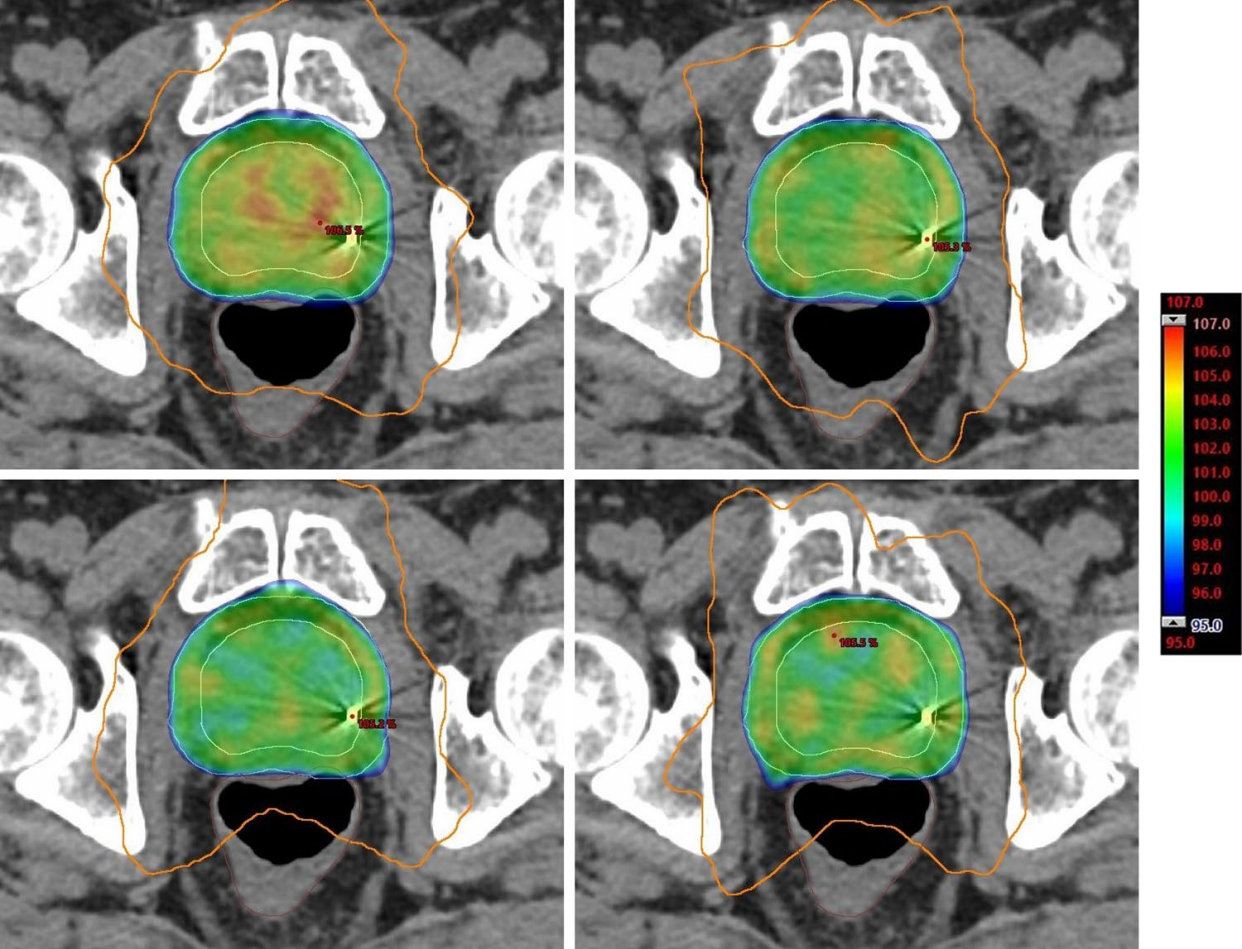
The dose distribution of a patient from Centre B representative of the majority of patients. PTV shown in pink, CTV shown in orange, 50% isodose shown as orange, 100% isodose shown as cyan and the colour wash indicating 95–107% dose. Top Left—Model A. Top Right—Model B. Bottom Left—UCSD. Bottom Right—UCSD edit

Model B achieved the lowest proportion of OAR planning goals at all centres while Model A, the UCSD model and the two UCSD edit models gave similar results. Figures 2 and 3 show the bladder and rectum V51.3% (equivalent to V40Gy for a 78 Gy prescription) DVH information for all centres and models. All models performed similarly with regard to the bladder doses. However, the dose to the rectum was lower with the UCSD model compared to both Models A and B (*P* < 0.001 for both Models A and B at Centres B and C and for Model B at Centre A).The UCSD Edit models also produced lower rectum doses than Model A and B. The increased rectal sparing of the UCSD model can be seen in Fig. 4 where the 50% isodose line covers less of the rectum posteriorly.

#### Centre A

Model A achieved the highest proportion of planning goals at Centre A. While PTV planning objectives were more likely to be meet by Model B, the lower performance against the OAR planning objectives meant that this model was unacceptable to Centre A. Conversely, the UCSD and UCSD Edit models provided better rectal sparing and met more of the planning goals but performed poorly against the PTV planning goals.

It can be seen from Figs. 1, 2, 3 and 4 that UCSD edit model managed to maintain the low rectal doses of the original model. However, Centre A felt that the PTV V95% dose coverage achieved was inferior, visible in Fig. 1, and they were not able to remedy this.

They noted that the UCSD model achieved lower rectal doses. Information about the plans used to create each model is included in Table 4 in Appendix. These data show that the mean rectum doses for the plans in the UCSD model are lower than the plans Model A and Model B leading to lower predicted DVH curves and a lower line dose optimisation objective. In addition, the UCSD model, which included a wider range of rectal volumes than those in Model A or B, contained a number of upper objectives on the rectum which were not present in Model A or B.

The staff at Centre A found that the sharing of models was beneficial and discussed all models in a multi-disciplinary meeting. They chose to continue improving their in-house model (Model A) informed by the results of this study.

#### Centre B

For Centre B, the UCSD edit model achieved the PTV and OAR planning objectives more consistently than any of the other models performed. Despite this, Centre B did not select to use this model clinically.

Centre B is the only centre with a mean PTV dose goal. While the UCSD edit model satisfied more of the PTV planning goals than the other models, this particular goal was not satisfied for any patients using the UCSD and the UCSD Edit Models. In comparison, Model B achieved this objective for 95% of patients and Model A achieved this objective for 50% of patients. It is possible that renormalising the plans could have resulted in more plans satisfying this planning goal while still achieving better OAR doses. However, the planner at Centre B also noted that the global maximum dose with the UCSD and UCSD edit models was often outside the CTV (seen in 95% of plans using the UCSD model and 90% of plans using the UCSD edit model). While not a documented planning goal, the planner preferred the maximum dose to be in the CTV and this was achieved more often with the model developed in their own centre, Model B, with only 15% of plans using Model B having a global maximum dose outside the CTV. For Model A, which also uses multiple target structures, 75% of plans had the maximum dose outside the CTV.

#### Centre C

Model A achieved the most local planning goals at Centre C. The UCSD model fared poorly on the PTV planning goals and Model B was not able to satisfy as many OAR goals as Model A.

Based on their experience with the three models, Centre C expected that Model A would be the best model to adopt as their planning goals aligned better with Centre A, compared with Centre B. A lack of physics resources meant that the implementation of Model A was delayed and during this time an in-house model was developed by planning staff which was eventually implemented. The building of the in-house RapidPlan model at Centre C was informed by the other 3 models and hence participating in this project was considered beneficial.

Centre C reported that the sharing of models allowed them to become familiar with the software and to build a set of prostate plans to use in their own model. One of the main challenges at Centre C was finding agreement between their oncologists on the planning goals they should aim for. Centre C have implemented RapidPlan models for other treatment sites following this work.

## Discussion

Creating a RapidPlan model requires multiple good quality treatment plans. Using an externally generated model has the potential to produce superior plans while reducing the repetition of work already performed by others. This study investigates this further by comparing in-house RapidPlan models with externally developed models. Our results, from both centres A and B, show that their in-house models produce plans which are more likely to satisfy the local planning goals compared to externally produced models. This is not surprising given these models were specifically created with the planning goals of their centres in mind.

However, the assessment of models based on the performance of plans against local planning goals was incomplete. This style of assessment failed to highlight the reductions in rectal dose achieved by the UCSD model and other considerations such as location of global maximum dose.

Knowledge-based planning, as implemented in RapidPlan, aims to predict the optimal OAR DVHs and, based on these, set realistic optimisation criteria. As the UCSD model creates plans with lower rectal doses, it could be argued that this is the model which should be adopted for planning at the three centres. However, it may be that the lower rectal dose predictions given by this model are not compatible with the PTV requirements of the centres involved and, even with editing, the UCSD model could not produce plans clinically acceptable at the centres.

The failure of either Centre A and B to create a satisfactory model from the UCSD model may indicate adjustment of a model created with different aims is limited. Consequently, the centres may be biased towards their own in-house models and were not invested in further developing the external model.

Also, the adjustments to the UCSD model were limited by the single target structure used in this model. While additional target structures can be added to a model for each patient, the addition of an extra target by default for all patients invalidates the model within Eclipse and renders it unusable. All centres in this study found the PTV doses from the UCSD model were unsatisfactory and, without additional control provided by an extra target structure, were not able to adjust the model to produce satisfactory plans.

The lower rectal doses achieved with the UCSD model indicate a potential for improvement of the in-house models. Utilising additional fixed objectives on the rectum, when producing plans in the future, may produce lower rectal DVH curves. New iterations of models using plans with lower rectal DVH curves would lead to lower model predicted DVH curves and the production of plans with lower rectal doses.

While the in-house models were selected for use by all centres, these results may not be true for other comparisons where the shared model is better suited to the centre involved and to customisation. In addition, situations exist where generating a local model may not be feasible e.g. if patient numbers are low or planning practises have recently changed (ie. using a new planning system or changing from an IMRT to VMAT technique).

This study has highlighted the differing planning goals which exist between centres but the data also shows that the same model can give different results at different centres. Our results show that, for all models, the bladder doses are lower at Centre C, that the rectum doses are higher at Centre B and that PTV coverage is lower at Centre A. The differences observed could be caused by differences in contours or differences in planning techniques. As all centres chose to use a 2-arc planning technique, this is unlikely to be the cause. Centres B and C used 6MV whereas Centre A used 10MV. This may contribute to the higher rectum doses at Centre B but higher doses were not seen at Centre C using the same energy, and this does not explain the lower bladder doses at Centre C or lower PTV coverage at Centre A. A review of the rectal contouring guidelines at the three centres shows that these are not well documented at Centre B. This may be contributing to the higher rectal doses. Additionally, the patients’ prostate size as well as bladder and bowel preparation may also differ which could result in differing OAR and PTV contours.

While the shared models were not implemented clinically by the centres involve in this investigation, benefits were seen from the assessment of different models. All centres were able to benchmark in-house models against external models, determine areas of potential improvement for future model creation and learn by viewing the objectives set in other models.

Based on this work, an option to improve the success for future sharing of RapidPlan models and similar collaborative efforts is to work towards better agreement between centres, whereby planning goals and contouring guidelines are unified, prior to attempting to create a model. This option has several advantages in that it would incorporate external review of planning goals between centres, promote collaboration and discussion between centres and simplifies the task of creating a satisfactory model. This approach has been used successfully to create regional models in Germany and Victoria [8, 10]. In the Victorian model, plans from all centres were included in the creation of the model. This should ensure that the geometric range of patients at all centres is included. However, these models may need to exclude patient cohorts where systematic differences exist between target and OAR segmentations (eg potentially stratifying patients by use SpaceOAR gel to increase the space between the posterior prostate and rectum). When planning aims are agreed a-priori, for example in the context of a clinical trial, a share RapidPlan model has the potential to harmonise plan quality between centres and improve trial protocol compliance.

It has been seen in this study that considering only predefined planning metrics (i.e. DVH planning constraints) is not the only method used to assess plan quality. Even with prior agreement on planning goals, additional organ sparing following the as-low-as-reasonably-achievable (ALARA) [13] principal or improved PTV coverage may be achievable. The relative prioritisation and importance of these potential improvements may differ between radiation oncologists.

The success of sharing of RapidPlan models could be further improved by ensuring planning goals contained in the model are defined, and flexibility is built within the model. These features would allow wider sharing of models and would not require agreement or discussion prior to the creation of models. Centres could select a model which is most likely to align with their planning priorities and adapt this model in response to future changes in practice. In the specific context of RapidPlan, ensuring flexibly in shared models would mean including multiple PTV structures and a wide range of OAR structures to allow for the variation in planning goals between centres. This option would still require DVH predictions to be achievable in addition to the other added constraints, and hence all models need to be validated at the local level prior to clinical implementation.

A limitation of this study is that it only considered prostate radiotherapy. However, a comparison between an in-house created model and available shared models is likely to be beneficial for all treatment sites and ensure to best available RapidPlan model is used. Where the creation of an in-house model is not practical a flexible, shared model from a centre with similar planning goals is likely to be beneficial. However, internal testing and adjustment of the model are likely to still be required.

## Conclusions

When comparing radiotherapy plans generated using an in-house model with an externally generated model, it was found that the in-house model would most likely achieve the local planning goals and satisfy the requirements of the radiation oncologist. When an in-house model is not available, the model most likely to be considered acceptable is a model that has been created by a centre with similar planning goals. While the UCSD prostate model was found to achieve the lowest rectal doses, a lack of flexibility in adjusting the model meant that it could not provide plans which were satisfactory to any of the centres.

Participating in a comparison of RapidPlan models was found to be beneficial for the centres involved, even when the centre chose not to adopt the new models for patient planning. Where possible, centres should consider a range of RapidPlan models to find the model that best suits their needs.

When developing RapidPlan models for sharing or for internal use, models should be designed to be flexible, in particular, multiple target structures should be included as well as all available OAR structures. This allows more adjustment of the model where additional boost regions or additional OAR limitations are required. Model documentation should include details of the planning goals and beam arrangements of the plans used to create the model. This allows centres to select the most compatible models and then adapted these models to different planning constraints or techniques. The success of shared RapidPlan models may be increased through standardisation of planning goals and contouring guidelines as this will ensure more consistently DVH predictions.

## Appendix

See Table 4

**Table 4:** Data from the three models from the Varian Model Analytics program showing the optimisation objectives as well as minimum (min), mean, maximum (max) and standard deviation for the target and organs-at-risk both volumetrically and dosimetrically

	Model A	Model B	UCSD
Optimisation objectives			
PTV			D10% < 102% Priority 100
	D0% < 104% Priority 150	D0% < 102.5% Priority 110	D0% < 103% Priority 150
	D100% > 98% Priority 140	D100% > 100% Priority 130	D100% > 97% Priority 150
			D98% > 100% Priority 150
		D95% < 100% Priority 0*	
CTV or PTV inner		D0% < 106.5% Priority 140	
		D15% < 105% Priority 90	
	D100% > 101% Priority 160	D100% > 103% Priority 130	
Bladder	V99% < 0% Priority 90	V103.5% < 0% Priority 120	V95% < GV Priority 80
			V50% < GV Priority 80
			V25% < GV Priority 30
	Line Priority 60	Line Priority 65	Line GP
Femur_L/Femur_R			Line GP
Femoral Heads	Line Priority 60		
Penile Bulb			Line GP
Rectum	V99% < 0% Priority 90	V102% < 0% Priority 80	V100% < 0% Priority 150
			V95% < GV Priority 100
			V75% < GV Priority 90
			V50% < GV Priority 90
			V25% < GV Priority 80
			V10% < GV Priority 50
	Line Priority 60	Line GP	Line Priority 70
Input plan data			
Number of Targets	2	3	1
Number of OARs	3	3	5
Total number of plans	93	41	105
Targets	CTV	PTV inner	PTV
	PTV_High	PTV optimise	
		PTV low	
OARS	Bladder	Bladder	Bladder
	Rectum	Rectum	Rectum
	Femoral Heads	Femoral Heads	Femur_L
			Femur_R
			Penilebulb
PTV Target	PTV_High	PTV optimise	PTV
Volumetric [cm^3^]			
Min	70.39	7.44	44.55
Max	402.73	462.92	387.32
Mean	163.93	197.45	135.54
Std	70.46	82.42	53.33
Mean Dose [Gy]			
Min	32.59	45.07	9.15
Max	79.59	80.77	83.71
Mean	67.25	76.83	55.37
Std	8.24	7.83	26.17
Mean Dose [%]			
Min	99.32	101.89	101.08
Max	102.77	103.55	103.56
Mean	101.09	102.67	101.86
Std	0.67	0.44	0.47
Central Target	CTV	PTV inner	
Volumetric [cm^3^]			
Min	17.95	22.23	
Max	146.62	191.43	
Mean	54.59	69.47	
Std	21.50	37.21	
Mean Dose [Gy]			
Min	32.87	45.76	
Max	80.41	81.36	
Mean	68.17	77.52	
Std	8.40	8.11	
Mean Dose [%]			
Min	101.35	95.42	
Max	104.28	104.31	
Mean	102.46	102.98	
Std	0.64	1.42	
Bladder			
Volumetric			
Total min [cm^3^]	68.71	42.24	76.72
In-field min [cm^3^]	30.11	17.49	55.26
In-field min [%]	19.57	33.72	16.43
Out-of-field min [cm^3^]	0	0	0
Out-of-field min [%]	0	0	0
Overlap with target min [cm^3^]	1.84	2.61	2.9
Overlap with target min [%]	1.04	2.88	1.16
Total max [cm^3^]	632.26	546.2	685.52
In-field max [cm^3^]	261.84	358.76	555.6
In-field max [%]	87.25	94.93	94.15
Out-of-field max [cm^3^]	249.11	162.08	437.94
Out-of-field max [%]	52.2	45.37	66.49
Overlap with target max [cm^3^]	137.44	65.46	42.64
Overlap with target max [%]	60.63	66.28	22.7
Total mean [cm^3^]	236.83	192.06	265.39
In-field mean [cm^3^]	112.08	133.92	145.72
In-field mean [%]	53.23	71.2	60.75
Out-of-field mean [cm^3^]	41.2	26.95	50.41
Out-of-field mean [%]	11.77	10.5	13.25
Overlap with target mean [cm^3^]	24.4	20.56	13.04
Overlap with target mean [%]	12.04	13.8	5.67
Total std [cm^3^]	125.16	105.86	142.72
In-field std [cm^3^]	43.56	69.86	71.01
In-field std [%]	18.12	13.92	19.45
Out-of-field std [cm^3^]	64.59	40.20	88.80
Out-of-field std [%]	15.52	12.94	17.53
Overlap std [cm^3^]	22.47	12.17	8.95
Overlap std [%]	10.51	11.93	3.99
Mean Dose [Gy]			
Min	7.12	16.25	1.59
Max	58.99	60.66	40.51
Mean	26.10	38.23	16.93
Std	13.05	11.58	10.85
Mean Dose [%]			
Min	11.89	20.84	7.39
Max	89.38	81.97	55.77
Mean	38.92	50.99	29.83
Std	18.26	14.78	11.25
Rectum			
Volumetric			
Total min [cm^3^]	27.65	34.36	34.27
In-field min [cm^3^]	21.76	28.31	19.47
In-field min [%]	61.59	63.95	37.76
Out-of-field min [cm^3^]	0	0	0
Out-of-field min [%]	0	0	0
Overlap with target min [cm^3^]	0	0.22	0
Overlap with target min [%]	0	0.44	0
Total max [cm^3^]	111.58	145.84	226.16
In-field max [cm^3^]	101.4	108.79	134.92
In-field max [%]	97.79	94.83	98.35
Out-of-field max [cm^3^]	8.51	38.55	81.98
Out-of-field max [%]	18.67	26.44	46.33
Overlap with target max [cm^3^]	13.87	23.86	8.99
Overlap with target max [%]	22.25	29.98	12.61
Total mean [cm^3^]	56.19	69.68	74
In-field mean [cm^3^]	47.53	57.07	55.92
In-field mean [%]	84.82	83.41	78.22
Out-of-field mean [cm^3^]	0.36	4.24	6.28
Out-of-field mean [%]	0.57	4.73	6.47
Overlap with target mean [cm^3^]	4.85	6.5	2.76
Overlap with target mean [%]	8.6	9.46	3.97
Total std [cm^3^]	18.3	27.57	31.6
In-field std [cm^3^]	15.92	20.26	20.44
In-field std [%]	6.89	8.94	13.93
Out-of-field std [cm^3^]	1.39	8.37	13.58
Out-of-field std [%]	2.35	6.82	9.65
Overlap std [cm^3^]	3.24	4.3	1.75
Overlap std [%]	5.25	5.63	2.49
Mean Dose [Gy]			
Min	18.59	18.75	3.7
Max	44	48.63	36.24
Mean	32.43	42.44	19.02
Std	6.73	5.58	10.06
Mean Dose [%]			
Min	30.99	42.62	16.83
Max	66.01	70.2	48.58
Mean	48.64	56.2	34.27
Std	8.3	5.75	6.84
Femoral Heads/Femur			Left, Right
Volumetric			
Total min [cm^3^]	50.41	113.12	46.38, 46.26
In-field min [cm^3^]	41.42	102.78	32.17, 31.71
In-field min [%]	57.38	59.55	48.84, 49.37
Out-of-field min [cm^3^]	0	0	0, 0
Out-of-field min [%]	0	0	0, 0
Overlap with target min [cm^3^]	0	0	0, 0
Overlap with target min [%]	0	0	0, 0
Total max [cm^3^]	120.6	291.3	269.29, 266.21
In-field max [cm^3^]	120.52	271.62	196.92, 211.32
In-field max [%]	100	100	100, 100
Out-of-field max [cm^3^]	15.76	47.5	73.47, 75.49
Out-of-field max [%]	16.47	26.21	27.28, 28.36
Overlap with target max [cm^3^]	0	0	0, 0
Overlap with target max [%]	0	0	0, 0
Total mean [cm^3^]	77.94	177.92	69.02, 69.85
In-field mean [cm^3^]	74.63	147.54	63.87, 64.8
In-field mean [%]	95.97	83.39	94.49, 94.63
Out-of-field mean [cm^3^]	0.51	8.57	2.29, 2.14
Out-of-field mean [%]	0.58	4.53	1.54, 1.41
Overlap with target mean [cm^3^]	0	0	0, 0
Overlap with target mean [%]	0	0	0, 0
Total std [cm^3^]	14.88	27.4	35.91, 35.1
In-field std [cm^3^]	15.05	23.08	26.65, 26.06
In-field std [%]	8.56	8.47	10.53, 10.54
Out-of-field std [cm^3^]	1.95	10.69	10.79, 10.74
Out-of-field std [%]	2.25	5.37	5.29, 5.05
Overlap std [cm^3^]	0	0	0, 0
Overlap std [%]	0	0	0, 0
Mean Dose [Gy]			
Min	4.85	5.18	1.32, 1.17
Max	32.57	29.25	27.99, 24.05
Mean	17.58	17.17	12.03, 11.95
Std	6.17	4.61	7.36, 7.27
Mean Dose [%]			
Min	7.39	6.64	5.21, 3.28
Max	49.34	38.53	35.74, 32.85
Mean	26.37	23.19	21.88, 21.75
Std	8.54	6.52	6.99, 6.94

*G* generated, *D* dose, *V* volume, *P* priority

^a^Objective affects normal tissue objective
